# Unveiling the role of IL7R in metabolism-associated fatty liver disease leading to hepatocellular carcinoma through transcriptomic and machine learning approaches

**DOI:** 10.1007/s12672-025-02638-5

**Published:** 2025-05-23

**Authors:** Priyadharshini Annadurai, Arnold Emerson Isaac

**Affiliations:** Bioinformatics Programming Laboratory, Department of Bioscience, School of Bio Science and Technology, Vellore Institute of Technology, Katpadi, Vellore - 632014, Tamil Nadu India

**Keywords:** Metabolic-associated fatty liver disease, Hepatocellular carcinoma, Steatosis, Protein–protein interaction, Hepatic stellate cells, Cytokine signaling

## Abstract

Dysregulation of hepatic metabolism is a crucial factor in the development of fatty liver disease and significantly increases the risk of hepatocellular carcinoma (HCC). This study aims to identify the genes implicated in the prognosis of HCC among individuals suffering from metabolic fatty liver disease. We analysed protein–protein interaction (PPI) networks and constructed a  weighted gene co-expression network analysis (WGCNA) using  high-throughput gene expression profiling datasets. Our meta-analysis uncovered 442 differentially expressed genes (DEGs), comprising 30 upregulated and 412 downregulated genes. We constructed a PPI network from the DEGs and identified significant hub genes based on their degree centrality scores. Additionally, WGCNA highlighted impactful genes and tightly correlated modules, leading to the creation of a gene interaction network specific to metabolism-associated fatty liver disease (MAFLD). Pathway analysis revealed the candidate regulatory gene interleukin-7 receptor (IL7R), which is involved in cytokine-mediated signalling across both interaction networks. Pro-inflammatory cytokines interact with IL7R, activating the JAK/STAT pathway that influences gene expression throughout progression to HCC. IL7R activates STAT3, affecting the behaviour of activated hepatic stellate cells following initial liver damage. Furthermore, the expression of the IL7R gene was validated as a predictor of HCC malignancy through a logistic regression model, resulting in an accuracy of 92%. Findings suggest that IL7R could be the target gene associated with metabolism-linked HCC. It could significantly impact the management of metabolic-associated fatty liver disease (MAFLD) and may help enhance HCC diagnostics to improve patient outcomes.

**Supplementary Information:**

The online version contains supplementary material available at 10.1007/s12672-025-02638-5.

## Introduction

Metabolic-associated fatty liver disease (MAFLD) prevalence has risen worldwide, making it the most common liver disease, affecting more than one-third of adults [1]. In 2020, a new term, MAFLD, was proposed for Non-alcoholic fatty liver disease (NAFLD), based on hepatic steatosis and at least one of type 2 diabetes mellitus (T2D), obesity, or metabolic dysregulation [2–4]. NAFLD is a chronic liver disease with no obvious cause, making it an exclusion diagnosis where other possible conditions were ruled out [5]. NAFLD affects 38% of the global population, with a growing risk of severe complications [6]. The disease is becoming more prevalent at an earlier age due to changes in diet, urbanization, obesity, and T2D. Also, there are some geographical disparities in NAFLD prevalence rates partly due to socio-economic and genetic factors [7]. Research has shown that NAFLD patients are more prone to develop hepatocellular carcinoma (HCC) [8, 9]. The burden of HCC is the third leading cause of cancer-related death, and one of the major causes is due to MAFLD [10–12]. Recently, MAFLD-related HCC has been reported more often, while viral hepatitis-related HCC has declined over the years [13]. Also, it disrupts normal mechanisms of hepatic stellate cells (HSCs), which are crucial in liver injury repair [14]. NAFLD is becoming increasingly recognized due to its rising prevalence worldwide, greater awareness among healthcare professionals, and improved understanding of these disorders [15, 16]. Patients with NAFLD and alcoholic liver disease are at a higher risk of developing HCC compared to those with viral infections such as hepatitis B and hepatitis C [17].

The presence of lipid droplets or lipid bodies in the organelles of hepatocytes is a characteristic feature of NAFLD, which can progress to fibrosis and HCC [18]. Hepatic steatosis, which is characterized by the accumulation of fat in the liver, is the hallmark of NAFLD etiology, and it is an evolving condition that can progress to cirrhosis and liver cancer [19, 20]. Even patients with treated and maintained virological responses are at a higher risk of developing HCC due to steatosis. The risk could be up to six times higher in such cases [21]. Obesity, hyperinsulinemia, glycaemic index (GI), and diabetes are some of the common risk factors for both steatosis and liver cancer. These factors can alter liver microcirculation and inflammatory cytokines, leading to liver metastases [22, 23]. The procoagulant state in MAFLD patients increases with NAFLD severity, possibly due to liver fat content, inflammation, atherogenic dyslipidemia, obesity or insulin resistance [24]. Likewise, the intake of imbalanced macronutrients such as saturated fatty acids (SFA), trans fats, and simple sugars can contribute to the development of NAFLD, which can have detrimental effects on liver metabolism [25, 26]. Moreover, hepatic steatosis is also linked to increased post-operative morbidity and mortality in individuals undergoing liver surgery [27]. Additionally, alcohol consumption can lead to diverse types of alcoholic liver disease (ALD), including hepatic steatosis, alcoholic steatohepatitis (ASH), fibrosis, cirrhosis, and HCC [28, 29]. Although some studies have suggested a potential association between hepatitis C virus (HCV) and alcoholism with HCC development, the causal relationship between HCV and alcoholic HCC remains controversial [30].

The pathogenesis of NAFLD-related liver carcinogenesis is distinct and involves a complex interplay of multiple processes, including environmental factors, oxidative stress, chronic inflammation, and the immunological response. A meta-analysis reveals that NAFLD, characterized by abnormal liver enzyme levels and abdominal ultrasound, increases the risk of T2D and metabolic syndrome, necessitating non-invasive procedures for treatment [31]. MAFLD, when combined with other liver diseases, increases complications and mortality rates in patients, while NAFLD research focusing on hepatic phenotype is much needed [32]. Based on the literature survey, the present study identified the predictive biomarkers involved in developing HCC with steatosis. This study considered the development of HCC with fatty liver disease, metabolic disorders, alcohol abuse, and steatosis as underlying disorders that fulfil the MAFLD criteria [33, 34]. Transcriptomic data of the patients were used, and differential gene expression analysis was carried out after normalizing the samples. Functional enrichment and pathway analysis were performed to explore the function of DEGs. The protein–protein interaction (PPI) network was constructed to identify the hub genes. Additionally, weighted gene co-expression network analysis (WGCNA) was carried out with a validation dataset to find the module-trait relationship of the prominent biomarker genes. WGCNA helps researchers identify functional interpretation of the genes that is biologically meaningful in uncovering cancer progression processes, and correlate gene modules with clinical data for diagnostic and prognostic biomarkers, highlighting key regulatory genes as therapeutic targets [35–37]. In the WGCNA module, the co-expression network was built, and the correlation between each pair of genes was computed initially. Then, their distribution was fitted to a power law, and an adjacency score was calculated accounting for the network topology. Based on these approaches prominent biomarkers were identified for the prognosis of HCC underlying MAFLD.

## Methods

### Dataset acquisition

The study utilized two RNA-seq datasets that were obtained from the publicly available gene expression omnibus (GEO) database [38]. The search strategy was as follows: “fatty liver disease”, “homo sapiens” (organism), and “expression profiling by high throughput sequencing” (filter). The resulting entries were further screened based on the relevance to HCC, not exposed to viral infection and non-treatment-based studies such as drug treatments. The first dataset GSE140462 consisted of 14 samples, out of which 7 samples had a fatty liver background, and the remaining 7 were adjacent normal tissues of the patients [39]. The second dataset GSE184733 comprised 34 samples, of which 6 samples were considered for the study, containing 3 in each patient and normal samples, while the other samples were excluded due to viral infections [40].

### Gene expression fold-change analysis

To ensure accurate results, the study filtered out lowly expressed genes as they could increase false discovery rates (FDR) and limit the ability to detect differentially expressed genes (DEGs) while estimating the FDR, where the rate of those features called significant is truly null. The edgeR function was used to filter these genes based on minimum counts per million (CPM) thresholds [41]. Samples with CPM above 0.5 in at least two sample groups were retained. Normalization was performed to eliminate composition biases between samples and the quality control of the samples was further visualized using MDS plot and boxplot (Fig. S1). To prepare the data for differential gene expression analysis, the read counts were transformed into logCPM, taking into account the mean–variance relationship in the data [42]. After converting the voom transformation, the study used the limma function to analyze the DEGs. The genes were filtered based on the log₂ fold change (FC) cutoff of 1 and an adjusted p-value (Benjamin-Hochberg) less than 0.05 .

### Network analysis

The PPI network of the differentially expressed genes was constructed using the STRING database (https://string-db.org/) [43] with a confidence level of 0.4 and higher as a filter. The network was visualized using Cytoscape [44, 45]. The top 10 genes were identified based on their degree as hub genes using the Cytohubba plugin, as described by Bader & Hogue (2003) and Chin et al. (2014) [46, 47]. The degree centrality was calculated based on the number of neighbors a node has, and it determines the dominance of the network graph using Freeman’s general formula, indicating the degree of centralization [48, 49].

$${C}_{D}=\frac{\sum_{i=1}^{n}({k}_{max}-{k}_{i})}{(\text{n}-1)\,(\text{n}-2)}$$

where C_D_ is the degree centralization of the network, n determines the number of nodes, *k*_*i*_ defines the node *i*, and *k*_*max*_ is the maximum degree in the network, this formula calculates the sum of the difference between the maximum degree and the degree of each node, then normalize it by dividing by the maximum possible sum of differences. The sum considers degrees between the largest and given vertex, while the denominator normalizes results. The degree determines the number of interactions associated with the particular node, and hub genes have a large degree of interactions, and they are hypothesized to play an essential role in the network, which is associated with the biological process [50, 51].

### Co-expression network analysis

**Fig. 1:**
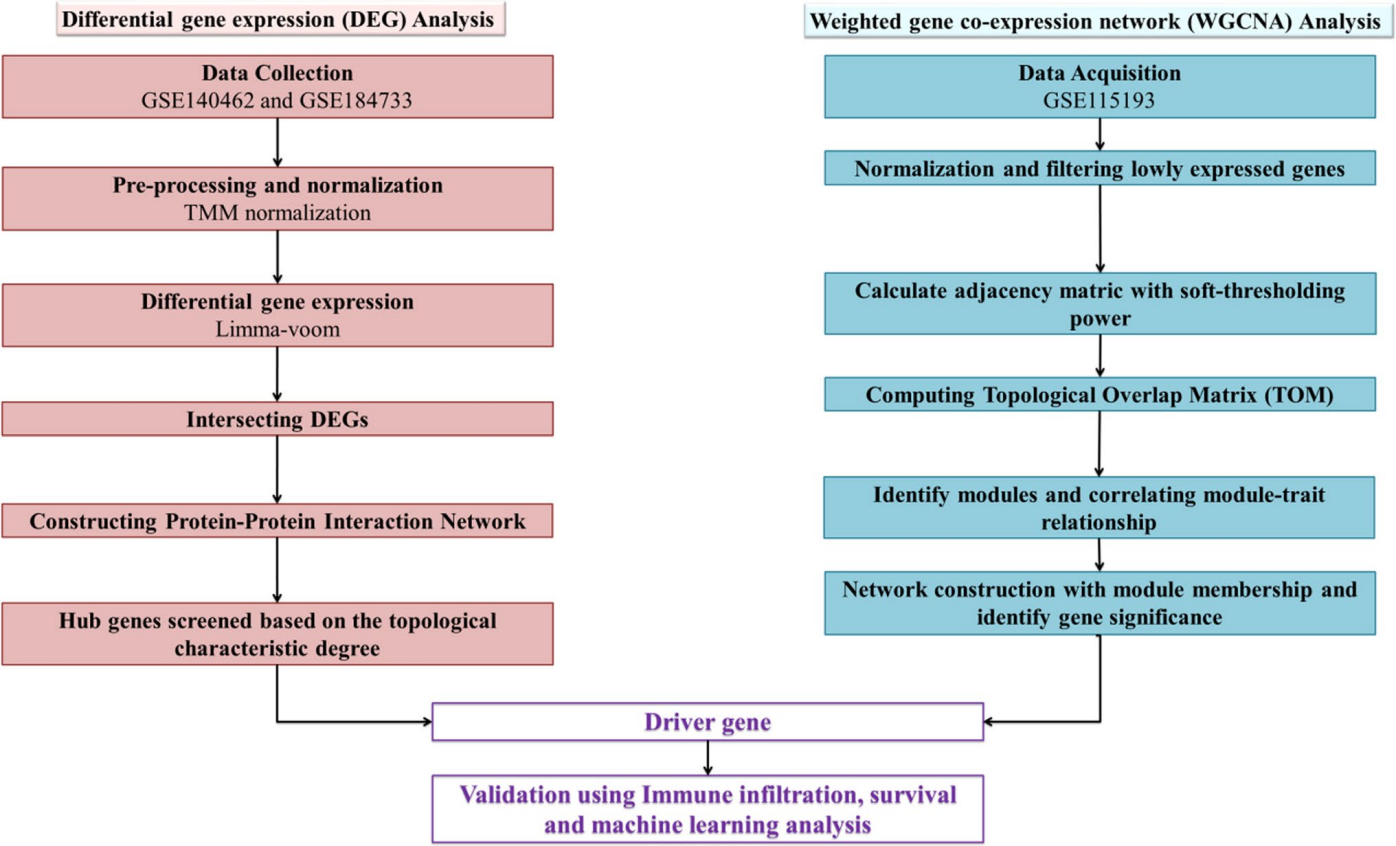
Study workflow of this integrated bioinformatics analysis

The study employed GSE115193, an independent validation dataset, to construct a gene co-expression network using the WGCNA (v 1.47) package in R [52]. After filtering lowly expressed genes and normalization, gene expression values were imported into WGCNA to construct co-expression modules using the automatic network construction function blockwiseModules with default settings, assigning the soft threshold power and Topological Overlap Matrix (TOM) Type was unsigned. Then, the Pearson correlation analysis was used to identify modules significantly associated with phenotypic characteristics [53]. Finally, the modules network was mapped using Cytoscape 3.7.2. The overall pipeline of the study has been shown in Fig. 1 to find the driver genes.

### Pathway enrichment analysis

Functional and enrichment analysis of the hub genes involved in the biological process, cellular components, molecular functions and Kyoto Encyclopedia of Genes and Genomes (KEGG) pathway analysis was carried out using the DAVID (Database for Annotation, Visualization and Integrated Discovery) online tool [54]. The Benjamin-Hochberg method was used to calculate the p-value [55].

### Validation of hub gene

The hub genes were further studied for immune infiltration and survival analysis. Also, logistic regression machine learning models were used to predict the HCC outcome using the hub gene expression pattern through the TCGA-LIHC dataset [56]. The logistic regression model is preferred for predicting binary outcomes, particularly gene expression levels, due to its ease of modelling, robustness, and ability to include categorical variables, even in small datasets [57]. The model was validated based on the 10-fold cross-validation, model performance, precision and recall.

## Results

### Differential gene expression analysis

**Fig. 2:**
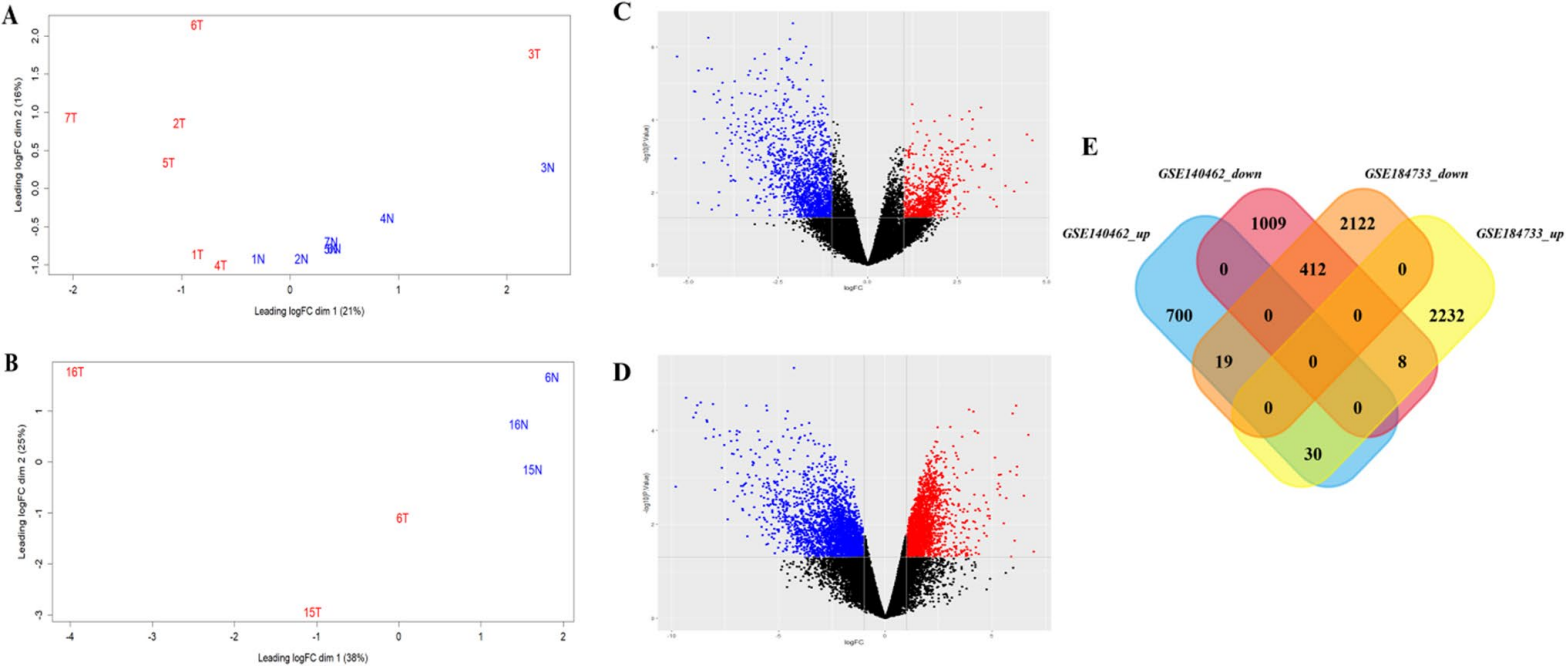
RNA-Seq differential gene expression. **A** MDS plot for GSE140462. **B** MDS plot for GSE184733 samples. T represents the cancer samples, and N represents the normal samples. **C** Volcano plot for GSE140462. **D** Volcano plot for GSE184733. **E** Venn diagram identify the intersecting DEGs in both datasets

After normalization, the variance between the sample groups has been studied. The Euclidean distances were calculated, and the distance between each pair of points represents the variance between the sample groups [58, 59]. The MDS plot shows the variance between the samples based on their dimensions, with the x-axis being Dimension 1 and the y-axis being Dimension 2. In Fig. 2A, dimension 1 is 21%, and dimension 2 is 16% in the samples from the GSE140462. Similarly, in Fig. 2B, dimension 1 of GSE184733 is 38%, and dimension 2 is 25%, which shows the variance among the sample group is lesser when compared to the first dimension. In this analysis, both datasets showed higher first-dimensional variance when compared to dimension 2.

After transformation, the genes are analyzed for differential expression using the Limma tool [60]. The empirical Bayes function was used to predict the up and down-regulated genes, with a fold change cutoff of 1 and a p-value less than 0.05 (Fig. 2C, D). This approach helps in identifying the genes that are significantly differentially expressed between the two datasets, which can be further studied to understand their role in the biological processes of interest. A Venn diagram approach was used to identify the common DEGs in both datasets [61]. The analysis revealed that 30 commonly up-regulated and 412 commonly down-regulated genes were expressed in both datasets (Fig. 2E). These genes were further subjected to protein–protein interaction (PPI) network analysis.

### Network analysis

**Fig. 3:**
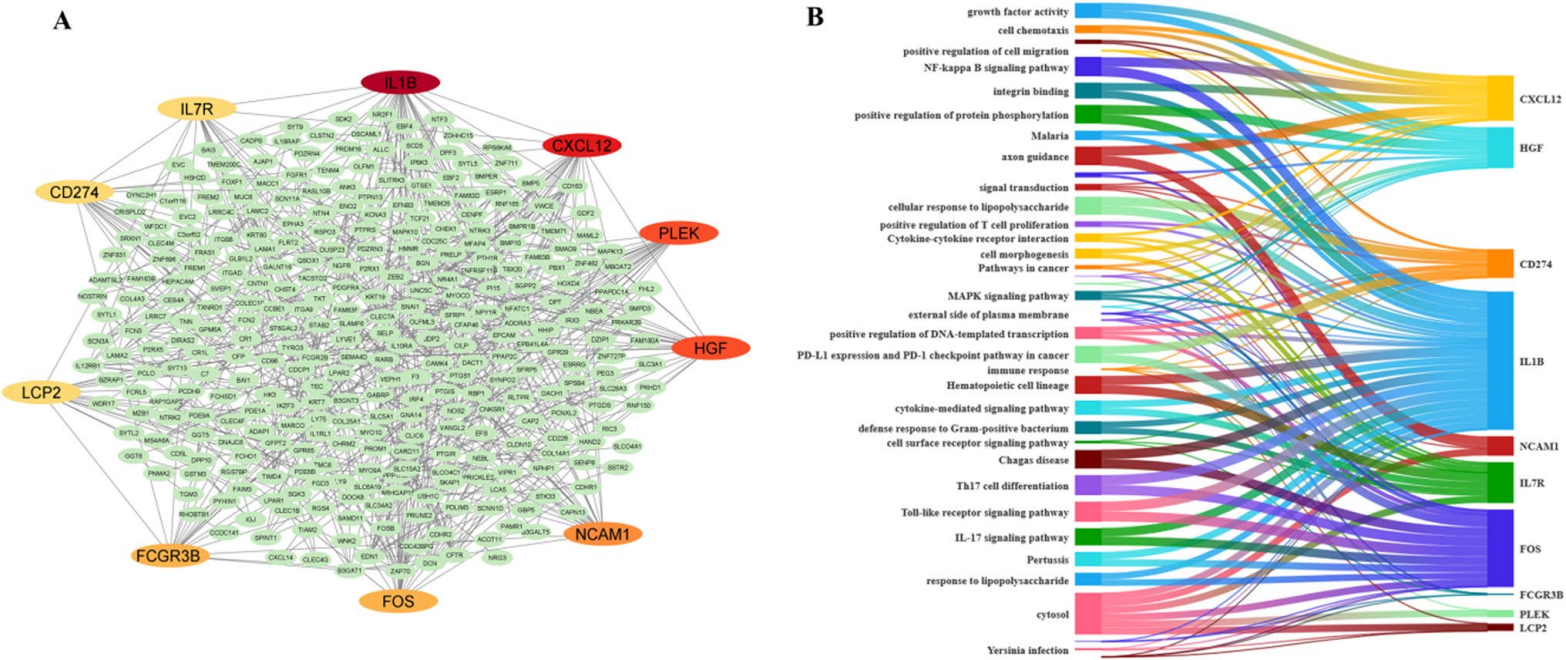
Hub gene analysis. **A** Protein-proteins interaction network and colored nodes represent the highlighted top 10 hub genes. **B** Enriched terms of the hub genes

A PPI network was constructed using 442 genes from the DEG analysis with the help of the STRING database. The resulting PPI network consisted of 338 nodes and 756 edges (Fig. 3A). The Cytohubba plugin was used to identify the top 10 hub genes based on their degree centrality as their topological characteristics (Table 1).

**Table 1:** Topology characteristics of hub genes

Gene	Degree centrality	Average shortest path length	Betweenness centrality	Closeness centrality	Clustering coefficient	Neighborhood connectivity	Radiality	Stress
IL1B	38	2.80	0.23	0.36	0.14	11.18	0.95	107,534
CXCL12	25	2.94	0.09	0.33	0.20	13.16	0.94	49,004
PLEK	23	3.56	0.09	0.28	0.10	6.34	0.93	40,948
HGF	23	3.12	0.06	0.32	0.20	12.43	0.94	46,164
NCAM1	22	3.02	0.12	0.33	0.23	13.32	0.94	52,216
FCGR3B	20	3.21	0.03	0.31	0.31	14	0.94	24,866
FOS	20	3.23	0.05	0.31	0.12	8.95	0.94	25,044
LCP2	20	3.49	0.02	0.29	0.21	10.4	0.93	23,244
CD274	20	3.16	0.03	0.32	0.34	15.05	0.94	21,042
IL7R	19	3.24	0.02	0.31	0.35	14.16	0.94	16,654

Based on Freeman’s algorithm, the hub genes were screened and highlighted in Fig. 3A. The hub genes resulted from the PPI network analysis include IL1B, CXCL12, PLEK, HGF, NCAM1, FOS, FCGR3B, LCP2, CD274 and IL7R. The enriched terms of the hub genes are shown in Fig. 3B**, **which are involved in the biological pathways. Also, the detailed description of the enrichment analysis has been represented in the Table.S2.

### Weighted gene correlation network analysis

**Fig. 4:**
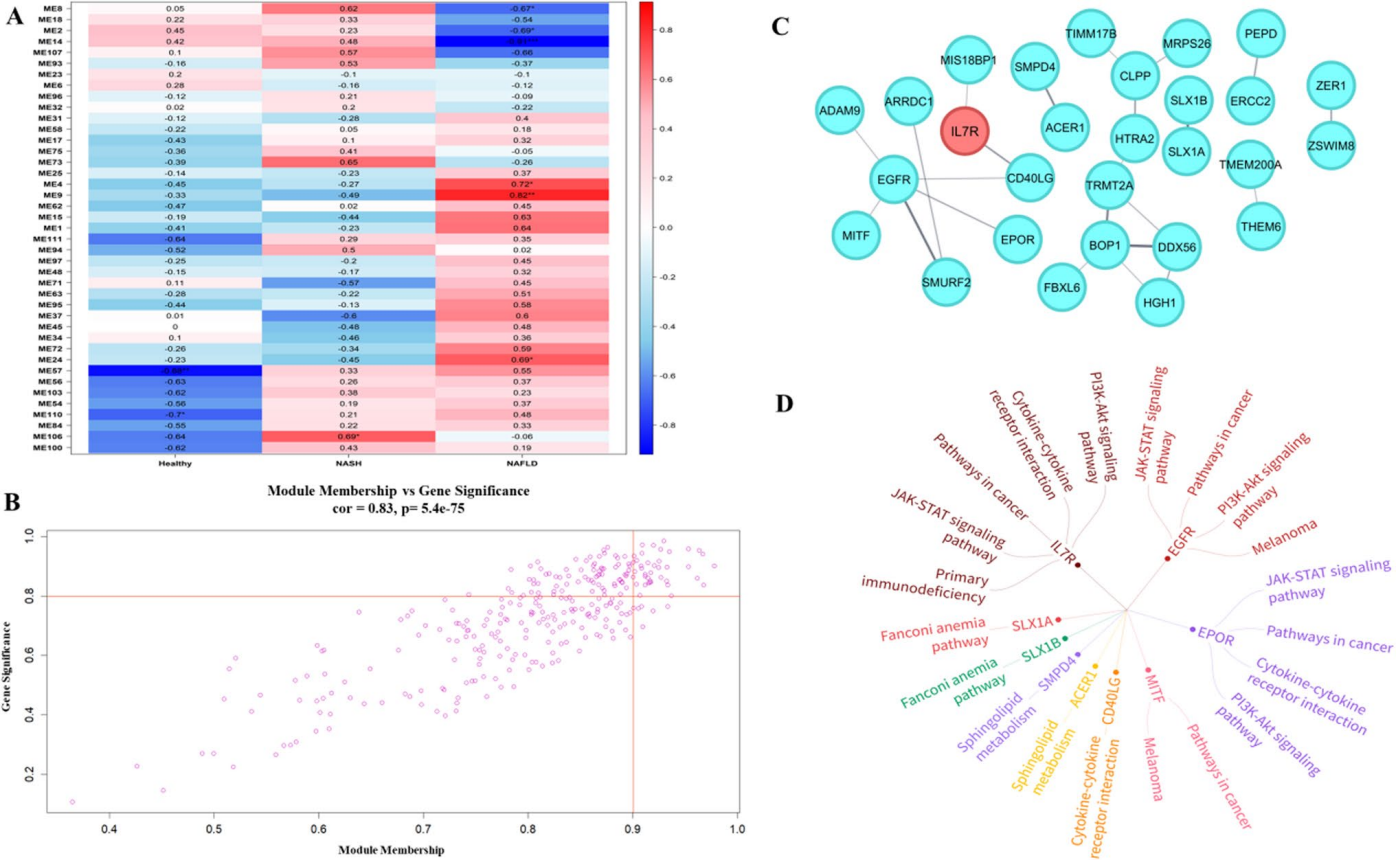
WGCNA module identification. **A** Heat map of module-trait association. **B** Correlation of module (ME14) with gene significance. **C** Gene interaction network analysis highlighting significant genes. **D** Enriched pathways of significant genes

The WGCNA was used to analyze the connection between genes and physiological traits, discovering the hub genes associated with physiological and biological traits. After filtering lowly expressed genes, expression values were imported to construct co-expression modules using the blockwiseModules function with a soft threshold power 10 (Fig. S2). Fig. S3 shows the cluster dendrogram of the genes after normalization of the validation dataset. As shown in Fig. 4A, we identified significantly associated modules with physiological or biological traits in NAFLD. ME14 (Module Eigen 14) module was significantly associated with the NAFLD trait. Accordingly, the correlation of the ME14 module with gene significance resulted in significance correlation of 0.83 (Fig. 4B). Based on this analysis, the co-expression network of ME14 module was further analyzed. We selected the genes with gene significance (GS) > 0.8 and module membership (MM) > 0.9 to construct the network regulatory map, and then highlighted gene significance associated with NAFLD trait (Fig. 4C). Pathway analysis of the significant genes has been carried out and shown in Fig. 4D**.** Most commonly enriched pathways include cytokine- cytokine receptor interaction pathway, JAK-STAT signaling pathway, PI3 K-Akt signaling pathway and pathways in cancer (Table S2).

## Discussion

Dysregulated metabolic pathways often act as a risk factor for cancer. The global incidence rate of MAFLD-related HCC has increased, distinct from other HCC etiologies, particularly in the absence of cirrhosis [62, 63]. Based on this research, our study involved the use of transcriptome datasets, which were obtained from the NCBI GEO database. The overview of the study is shown in Fig. 1. After preprocessing, the significant source of variation between the samples was visualized (Fig. 2A, B); these results are reflected in the number of DEGs obtained when comparing the samples from different groups as described by Law et al. [64, 65]. Volcano plot depicts the similarity of gene expression pattern from the MDS plot where the number of DEGs is more in the Fig. 2D, and the variance between the samples is higher in Fig. 2B when compared to Fig. 2C. Intersecting the DEGs resulted from both the dataset was 442 intersecting commonly expressed genes (30 up-regulated and 412 down-regulated) in comparison between the two transcriptome datasets (as shown in Fig. 2E). To evaluate the physical and functional associations among the DEGs, we utilized the PPI network using the STRING database [66]. PPI network was constructed using 442 DEGs as nodes, and genes without interactions were removed from the network, as shown in Fig. 3A. Hub genes were identified based on their topological characteristics; results are presented in Table 1. It includes average shortest path length, betweenness centrality, closeness centrality, clustering coefficient, neighborhood connectivity, radiality, and stress. This study also utilized functional enrichment analysis to identify overrepresented biological pathways in the hub genes [67, 68]. The resulting hub genes have been reported to be involved in the inflammation, immune response, cell signalling, proliferation, cell adhesion and interaction, immune evasion and transcriptional regulations in HCC development, as highlighted in Fig. 3A [69–71]. Also, these results are reflected in Fig. 3B, which illustrations that most of the hub genes are enriched in the immune response, regulation of plasma membrane; integrin binding, cytokine signalling pathways and regulation of immune cells.

**Fig. 5:**
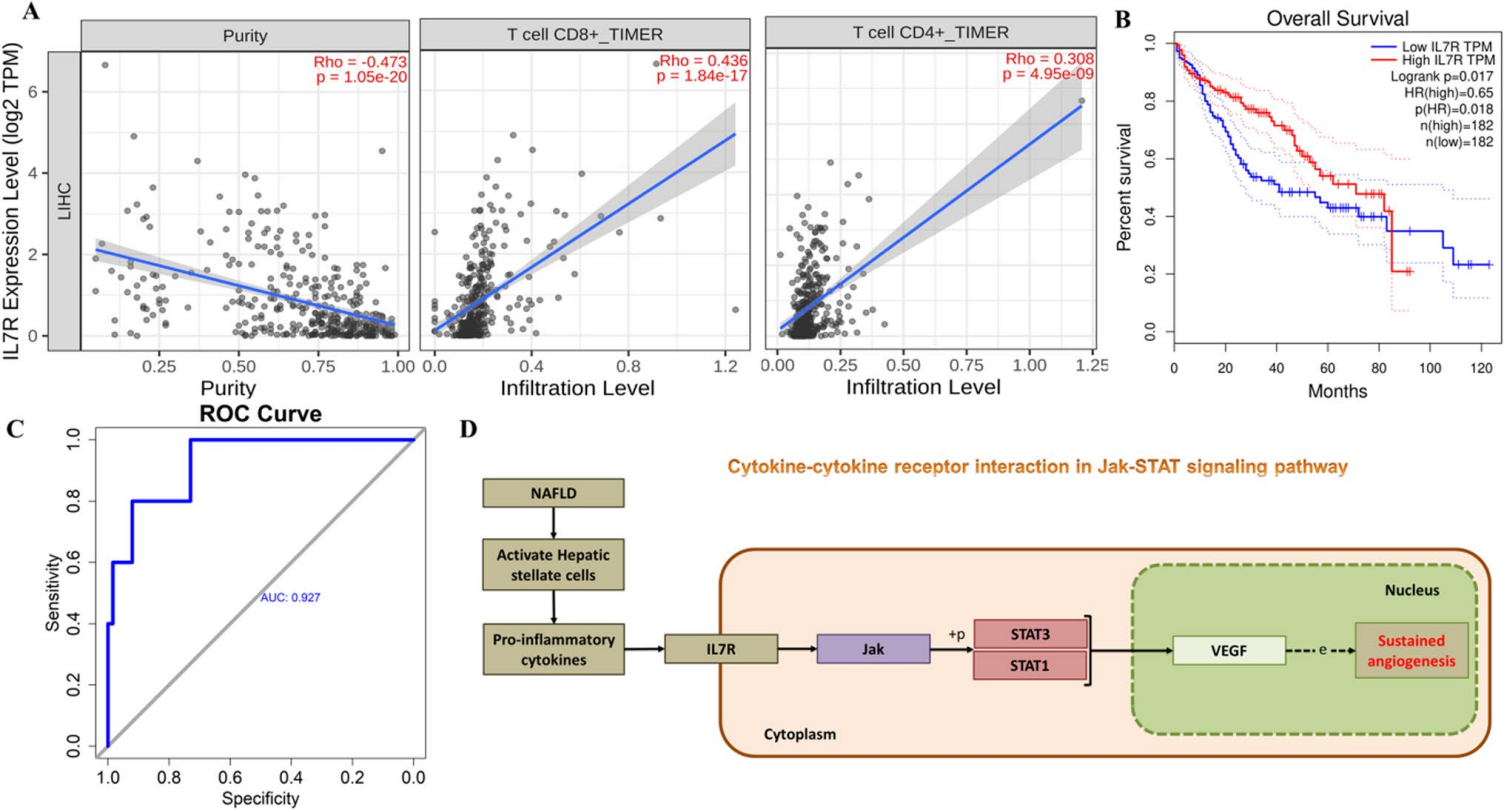
Validation of the hub gene. **A** Immune infiltration of T cells. **B** Survival analysis. **C** ROC curve for validating IL7R expression. **D** Mechanism underlying the transition of fatty liver disease to HCC

Subsequently, we approached WGCNA with an independent dataset to identify highly synergistic genomes and possible markers through an assessment of the interrelationship between genomes and their relationship to phenomena. For constructing co-expression modules we implemented blockwiseModules function with default settings, assigning the soft threshold power 10 and TOM Type is unsigned. By evaluating the interaction between each module and the molecular mechanism of cancer, the most prominent module ME14 was selected (Fig. 4A). The correlation of the module membership with gene significance is 0.83 (Fig. 4B). Gene interaction network was constructed based on the weighted genes in ME14 module (Fig. 4C). Enrichment pathway analysis of the significant genes has also been shown in Fig. 4D. From both the networks, it evidently shown that most significant genes are cytokines or cytokine receptors which are majorly involved in the immune system regulation (Figs. 3A and 4C). The pathway analysis also reflects the functionally enriched cytokine-cytokine receptor signaling cascades. Consequently, based on the network analysis resulted from PPI network (Fig. 3A) and WGCNA module (Fig. 4C), the IL7R gene was found to be involved in both the analysis related to traits and as well as hub gene from the transcriptome analysis. As shown in Figs. 3B and 4D the driver gene IL7R, a cytokine receptor has been majorly involved in the cytokine-cytokine receptor signaling. Enrichment analysis shows that it is IL7R is majorly related to cytokine signaling functions (Table S1). The JAK/STAT signaling pathway, particularly via IL7R, is a crucial link between MAFLD and HCC because it promotes tumorigenesis, cell survival, and proliferation, leading to malignant transformation. The JAK/STAT pathway is particularly relevant in HCC due to its direct involvement in oncogenic processes driven by chronic inflammation and metabolic dysregulation [72, 73]. Its activation mechanisms through cytokines are a critical target for therapeutic intervention compared to the more general roles of PI3K and MAPK pathways [74]. The activation of the IL7R-JAK-STAT pathway is linked to alterations in the tumor microenvironment, affecting immune cell infiltration and activity [75]. The immune infiltration analysis also shows that IL7R gene is negatively correlated with the tumor purity in LIHC samples in the immune cells CD8 + and CD4 + T cells (Fig. 5A). From KEGG analysis, IL7R interacts with JAK1 and JAK3 upon binding, activating JAK kinases and regulating gene expression [76]. Further validating the IL7R as a driver gene we utilized a logistic regression machine learning model to predict the malignancy of HCC based on the gene expression and sample pathology. It predicts the probability of observations belonging to different categorical outcomes and generalize linear model that relaxes assumptions, making it useful for non-continuous outcomes [77, 78]. We obtained an accuracy of 92% in predicting the HCC outcome in logistic regression machine learning model (Fig. 5C). Further, the model was evaluated for its performance through a precision-recall (PR) curve. From Fig. S4, we obtained an area under the curve (AUC) of 0.933 in the PR-curve analysis, which demonstrates that the model has a very high performance in distinguishing the positive and negative cases. In the same way, highly effective at identifying the positive instances while minimizing the false positives.

In the event of liver injury, HSCs are activated, leading to the transition from NAFLD to HCC, involving changes in the immune microenvironment, with increased infiltration in NAFLD and reduced cytotoxic T cells in HCC [79–82]. HSCs regulate hepatic homeostasis, maturation, and differentiation and are also involved in hepatic inflammation by releasing inflammatory cytokines and chemokines that interact with other liver cells [83]. IL-7, a pro-inflammatory cytokine released by the immune cells, binds to IL7R and involves the disease progression as shown in Fig. 5D [84–86]. Further, the IL-7/IL-7R axis activates the JAK/STAT pathway, specifically STAT3, which influences the HSC behavior [87, 88]. This axis is crucial in promoting T-cell development, differentiation, proliferation, and immune system reconstitution after depletion [89–93]. Polymorphisms in the IL7R gene are linked to liver stiffness, affecting the T-cell homeostasis and immune response in disease progression [94–96]. Immune infiltration analysis confirms T cell positive regulation in the HCC environment is correlated with enrichment analysis (ref Figs. 3B and 5A). IL7R gene expression changes influence macrophage interactions, epithelial to mesenchymal transition (EMT) promotion, T cell proliferation, and survival [97–99]. The immunosuppressive environment in HCC is characterized by reduced cytotoxic T cells and increased regulatory T cells, facilitating immune evasion and tumor progression. High IL7R expression in activated CD8 + T cells improves HCC patient survival [100–102]. In relation to that, our survival analysis also reflects the high-expression group has more survival than the low-expression groups as shown in Fig. 5B. Based on this evidence, activation of hepatic stellate cells triggers the IL-7/IL-7R axis, potentially reversing activation for disease management. To summarize, metabolic disorders cause lipotoxicity, leading to inflammatory responses in liver tissues. HSCs accumulate, triggering pro-inflammatory and pro-fibrogenic functions. IL7 binds to IL7R, triggering EMT. This process activates the transition of fatty liver disease to liver cancer majorly HCC involving immune microenvironment changes and promoting sustained angiogenesis. Targeting IL-7/IL-7R could offer a therapeutic approach for fibrosis-related liver diseases.

## Conclusion

Metabolic dysfunction is a major factor in HCC invasion and managing inflammatory responses. We utilized transcriptome HCC datasets and identified the top ten hub genes from the PPI network. In addition to that, WGCNA was studied, and a gene interaction network was constructed to find trait-based gene significance. IL7R was identified as a driver gene for MAFLD traits in HCC. Tumors often exploit immune pathways for survival; the underlying mechanism behind the disease progression is the accretion of hepatic stellate cells, activating pro-inflammatory and pro-fibrogenic functions. The pro-inflammatory cytokines bind to the IL7R and trigger the JAK/STAT pathway, promoting cancer progression. Mitigating the IL-7/IL-7R axis could enhance immune surveillance and provide a therapeutic approach to liver disease management. A machine learning model predicts HCC malignancy based on IL7R gene expression, offering valuable insights into treatment options and early cancer management. Moreover, it can also be a perception for the diagnosis of non-cirrhotic HCC, where the development of HCC occurs in the absence of cirrhosis. Therefore, investigating the targets of IL7R in this context has the potential to provide valuable insights into treatment options for at-risk patients. It can also contribute to early cancer management in patients with underlying metabolic disorders. Targeting this pathway could offer new strategies for treating HCC by addressing the underlying inflammatory processes contributing to tumor progression. Further research is needed to elucidate how various cytokines contribute to aberrant activation of this pathway in different HCC subtypes, particularly in chronic liver diseases like NAFLD and NASH. Also, understanding these interactions could reveal how JAK/STAT mediates tumor progression and therapy resistance, especially in cases where multiple pathways are activated simultaneously. The limitation of the study is that it provides theoretical validation but requires further experimental validation. It offers insight into the pathogenesis of MAFLD progression from steatosis to HCC in non-cirrhotic patients. The MAFLD-HCC epidemic has resulted in a worldwide healthcare problem. Current therapy approaches do not consider etiology, resulting in underdiagnosis. Existing therapies for MAFLD-HCC are safe. However, randomized controlled studies are required to clarify therapy efficacy and stratify individuals.

## Limitations

The study on MAFLD progression from steatosis to hepatocellular carcinoma (HCC) in non-cirrhotic patients has limitations, including a reliance on theoretical validation and the need for extensive experimental validation. The ongoing MAFLD-HCC epidemic is a global concern, with current therapeutic approaches often failing to account for underlying etiology, leading to under diagnosis and suboptimal treatment outcomes. Existing therapies have shown safety, but randomized controlled trials are needed to evaluate their efficacy and identify potential side effects. RCTs also help stratify patients based on clinical and genetic parameters, facilitating personalized therapeutic approaches. A comprehensive approach involving theoretical and experimental research, including clinical trials is needed to develop more effective and targeted treatments for MAFLD-HCC, addressing knowledge gaps and improving patient outcomes.

## Supplementary Information

Supplementary material 1

## Data Availability

The GEO database from NCBI (Gene Expression Omnibus database, https://www.ncbi.nlm.nih.gov/geo/) was used to access the GSE140462, GSE184733 and GSE115193 datasets.
