# Supplementary material for: Unveiling the role of IL7R in metabolism-associated fatty liver disease leading to hepatocellular carcinoma through transcriptomic and machine learning approaches
Source: Discov Oncol. 2025 May 23;16:873. doi: 10.1007/s12672-025-02638-5 (PMC12102058; doi:10.1007/s12672-025-02638-5)
Supplement: Supplementary file 1 — Supplementary material 1 [file 12672_2025_2638_MOESM1_ESM.docx]

Supplementary Material

1. **Supplementary figures**

**
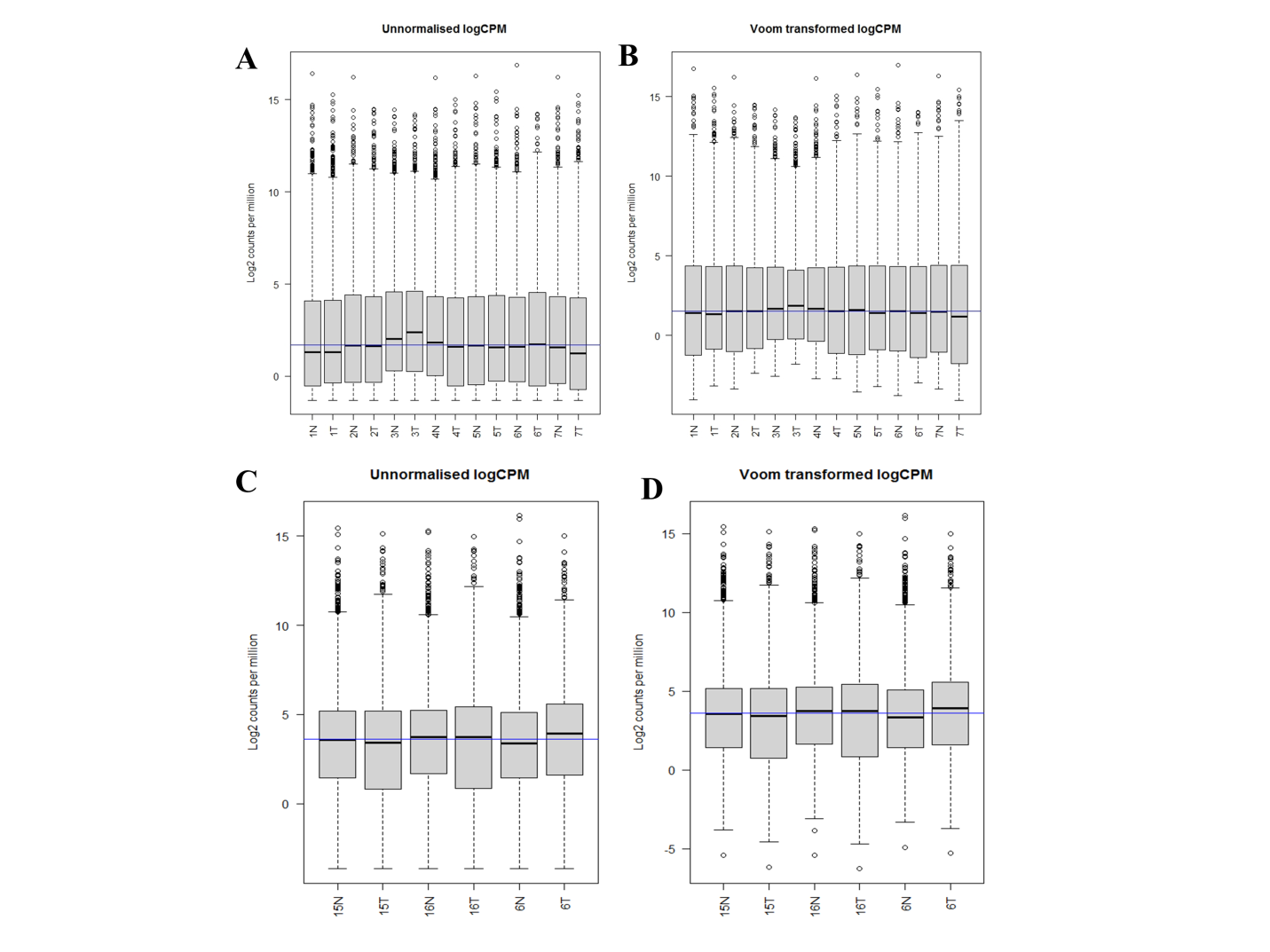
**

**Fig.S1:** Boxplot of the transcriptome dataset before and after voom normalization. (A, C) Normalization of GSE140462 dataset (B, D) Normalization of GSE184733 dataset

**
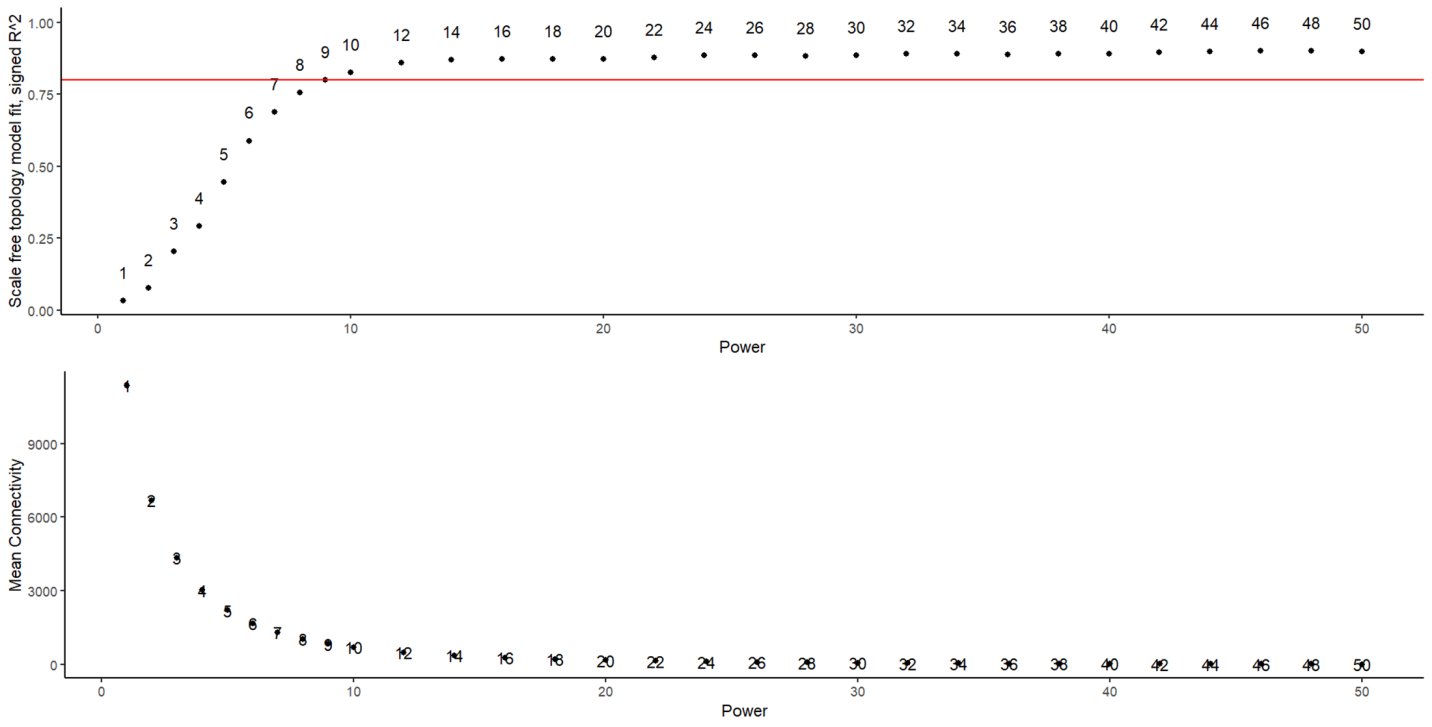
**

**Fig.S2:** Scale independence and mean connectivity for analyzing soft threshold power.


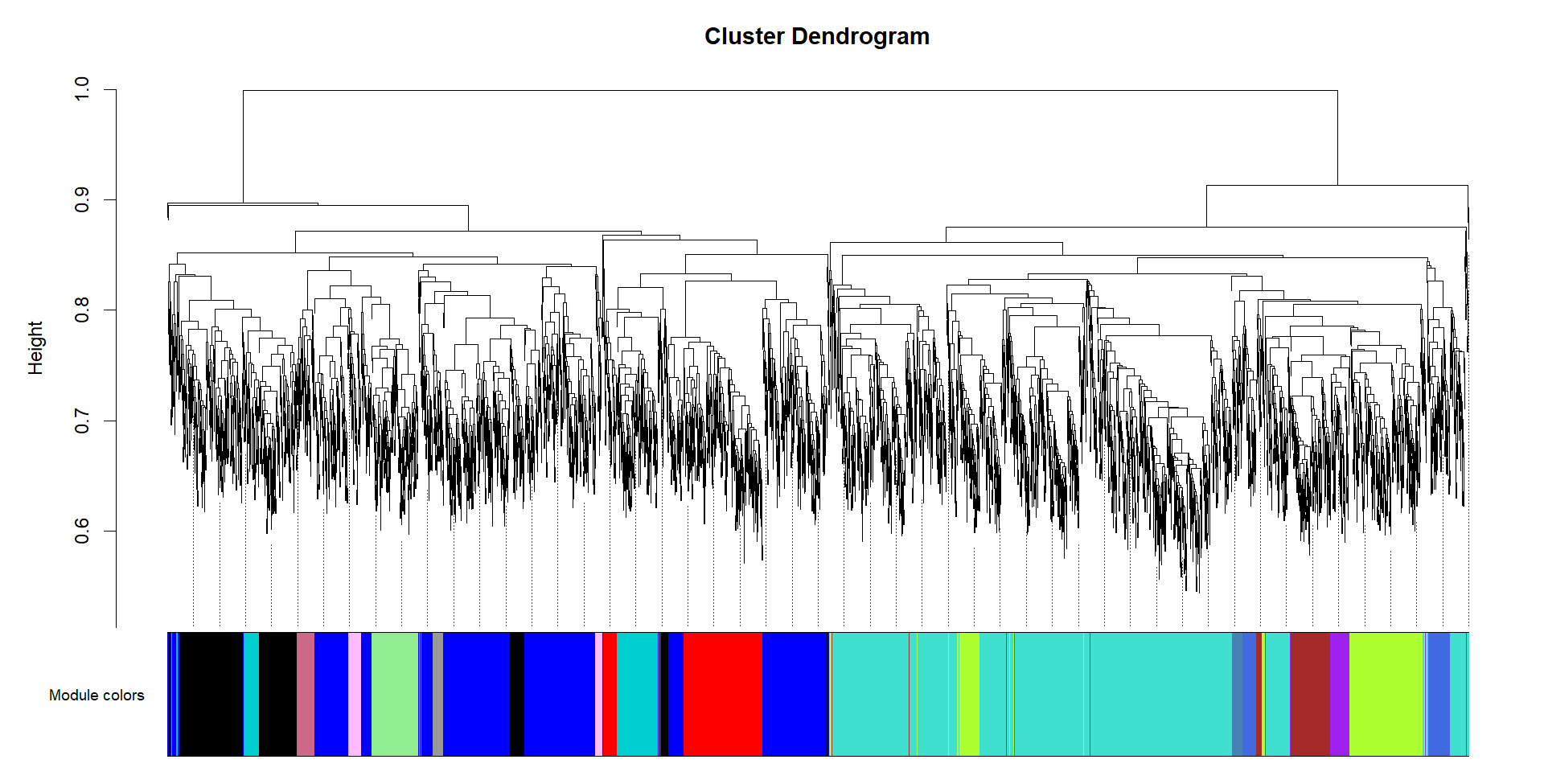


**Fig.S3:** Cluster dendrogram of the genes and modules colors.


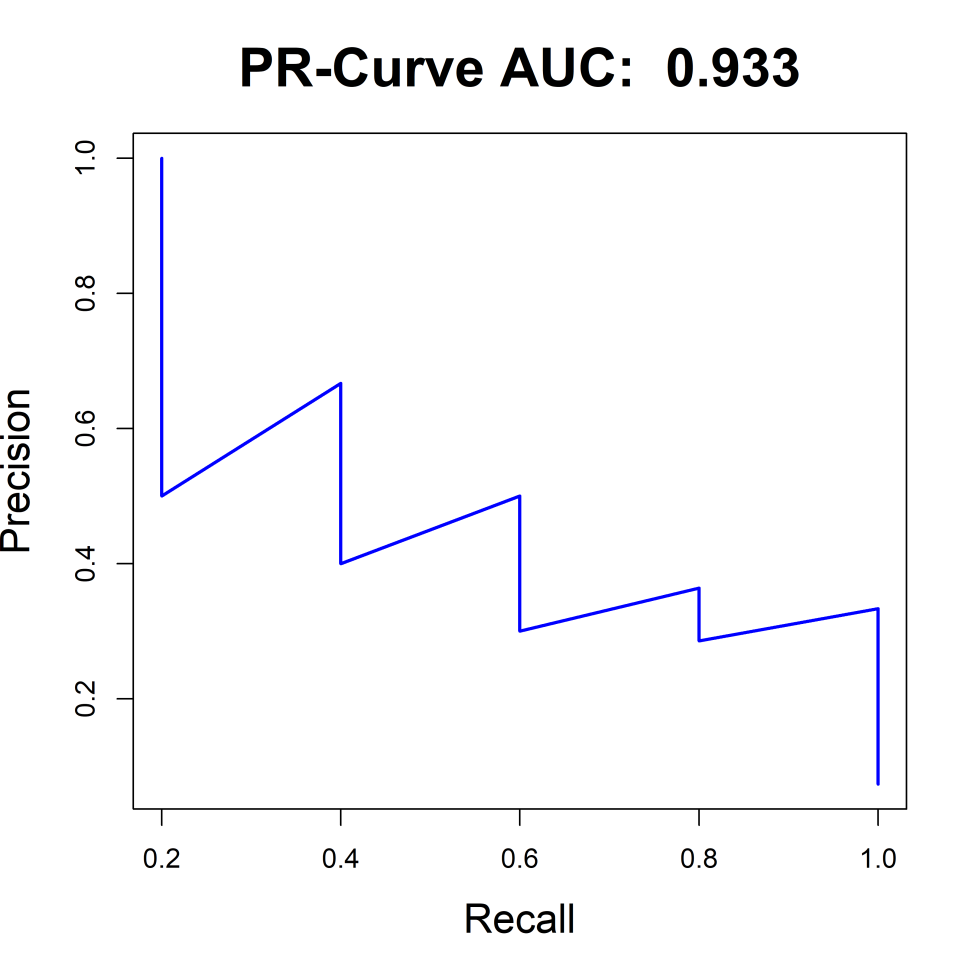


**Fig.S4:** Precision-Recall curve for the logistic regression model

1. Supplementary Tables

**Table.S1**: Enrichment analysis of Protein-protein interaction network hub genes

| **Category** | **Enrichment ID** | **Enriched Terms** | **P Value** | **Genes** | **Fold Enrichment** | **FDR** |
| --- | --- | --- | --- | --- | --- | --- |
| BP | GO:0006955 | immune response | 1.25E-06 | CD274, CXCL12, FCGR3B, IL1B, LCP2, IL7R | 23.44634 | 3.26E-04 |
| CC | GO:0009897 | external side of plasma membrane | 2.04E-05 | CD274, CXCL12, FCGR3B, NCAM1, IL7R | 24.34382 | 6.52E-04 |
| CC | GO:0005576 | extracellular region | 6.60E-05 | CXCL12, FCGR3B, IL1B, HGF, PLEK, NCAM1, IL7R | 6.95902 | 0.001057 |
| BP | GO:0030335 | positive regulation of cell migration | 2.40E-04 | CD274, CXCL12, IL1B, HGF | 27.50383 | 0.031393 |
| BP | GO:0014856 | skeletal muscle cell proliferation | 0.002734 | HGF, FOS | 657.8 | 0.238736 |
| BP | GO:0051450 | myoblast proliferation | 0.005914 | HGF, FOS | 303.6 | 0.387397 |
| BP | GO:0007166 | cell surface receptor signaling pathway | 0.008805 | CD274, FCGR3B, IL7R | 18.44299 | 0.461392 |
| BP | GO:0007165 | signal transduction | 0.015613 | CD274, CXCL12, IL1B, IL7R | 6.365806 | 0.681751 |
| BP | GO:0032733 | positive regulation of interleukin-10 production | 0.018996 | CD274, HGF | 93.97143 | 0.711005 |
| BP | GO:0001837 | epithelial to mesenchymal transition | 0.024365 | HGF, NCAM1 | 73.08889 | 0.79794 |
| BP | GO:0042102 | positive regulation of T cell proliferation | 0.027484 | CD274, IL1B | 64.70164 | 0.80009 |
| BP | GO:0045893 | positive regulation of DNA-templated transcription | 0.039649 | CD274, IL1B, FOS | 8.303226 | 0.960184 |
| BP | GO:0060326 | cell chemotaxis | 0.040313 | CXCL12, HGF | 43.85333 | 0.960184 |
| BP | GO:0000902 | cell morphogenesis | 0.046452 | HGF, IL7R | 37.95 | 1 |
| BP | GO:0050830 | defense response to Gram-positive bacterium | 0.058194 | IL1B, IL7R | 30.12824 | 1 |
| BP | GO:0032496 | response to lipopolysaccharide | 0.061648 | IL1B, FOS | 28.39424 | 1 |
| CC | GO:0005829 | cytosol | 0.066595 | IL1B, PLEK, LCP2, NCAM1, FOS, IL7R | 2.218481 | 0.710343 |
| BP | GO:0019221 | cytokine-mediated signaling pathway | 0.068523 | IL1B, IL7R | 25.46323 | 1 |
| MF | GO:0005178 | integrin binding | 0.073057 | CXCL12, IL1B | 23.8321 | 1 |
| MF | GO:0008083 | growth factor activity | 0.074364 | CXCL12, HGF | 23.39879 | 1 |
| BP | GO:0071222 | cellular response to lipopolysaccharide | 0.083828 | CD274, IL1B | 20.66387 | 1 |
| BP | GO:0001934 | positive regulation of protein phosphorylation | 0.090138 | IL1B, HGF | 19.15922 | 1 |
| BP | GO:0007411 | axon guidance | 0.090977 | CXCL12, NCAM1 | 18.975 | 1 |
| KEGG | hsa04380 | Osteoclast differentiation | 2.14E-04 | FCGR3B, IL1B, LCP2, FOS | 27.66823 | 0.021423 |
| KEGG | hsa05140 | Leishmaniosis | 0.002027 | FCGR3B, IL1B, FOS | 38.2684 | 0.100157 |
| KEGG | hsa05323 | Rheumatoid arthritis | 0.003005 | CXCL12, IL1B, FOS | 31.34752 | 0.100157 |
| KEGG | hsa05135 | Yersinia infection | 0.00637 | IL1B, LCP2, FOS | 21.35266 | 0.159241 |
| KEGG | hsa05200 | Pathways in cancer | 0.009714 | CXCL12, HGF, FOS, IL7R | 7.371274 | 0.194282 |
| KEGG | hsa04060 | Cytokine-cytokine receptor interaction | 0.027724 | CXCL12, IL1B, IL7R | 9.888143 | 0.401028 |
| KEGG | hsa04010 | MAPK signaling pathway | 0.028072 | IL1B, HGF, FOS | 9.822222 | 0.401028 |
| KEGG | hsa05144 | Malaria | 0.04438 | IL1B, HGF | 39.28889 | 0.554755 |
| KEGG | hsa05133 | Pertussis | 0.068473 | IL1B, FOS | 25.18519 | 0.574982 |
| KEGG | hsa05235 | PD-L1 expression and PD-1 checkpoint pathway in cancer | 0.078634 | CD274, FOS | 21.82716 | 0.574982 |
| KEGG | hsa04657 | IL-17 signaling pathway | 0.082839 | IL1B, FOS | 20.67836 | 0.574982 |
| KEGG | hsa04640 | Hematopoietic cell lineage | 0.086191 | IL1B, IL7R | 19.84287 | 0.574982 |
| KEGG | hsa05142 | Chagas disease | 0.089533 | IL1B, FOS | 19.07228 | 0.574982 |
| KEGG | hsa04064 | NF-kappa B signaling pathway | 0.091199 | CXCL12, IL1B | 18.70899 | 0.574982 |
| KEGG | hsa04659 | Th17 cell differentiation | 0.093694 | IL1B, FOS | 18.1893 | 0.574982 |
| KEGG | hsa04620 | Toll-like receptor signaling pathway | 0.094525 | IL1B, FOS | 18.02243 | 0.574982 |

**Table.S2**: Pathway enrichment analysis of the gene-gene interaction network

| **Category** | **Enrichment ID** | **Enriched Terms** | **P Value** | **Genes** | **Fold Enrichment** | **FDR** |
| --- | --- | --- | --- | --- | --- | --- |
| KEGG | hsa04630 | JAK-STAT signaling pathway | 0.020911 | IL7R, EGFR, EPOR | 12.14286 | 0.959569 |
| KEGG | hsa05200 | Pathways in cancer | 0.031868 | MITF, IL7R, EGFR, EPOR | 5.103189 | 0.959569 |
| KEGG | hsa05340 | Primary immunodeficiency | 0.050412 | CD40LG, IL7R | 35.78947 | 0.959569 |
| KEGG | hsa04060 | Cytokine-cytokine receptor interaction | 0.05982 | CD40LG, IL7R, EPOR | 6.845638 | 0.959569 |
| KEGG | hsa00600 | Sphingolipid metabolism | 0.070933 | ACER1, SMPD4 | 25.18519 | 0.959569 |
| KEGG | hsa03460 | Franconia anemia pathway | 0.072201 | SLX1B, SLX1A | 24.72727 | 0.959569 |
| KEGG | hsa04151 | PI3K-Akt signaling pathway | 0.084183 | IL7R, EGFR, EPOR | 5.635359 | 0.959569 |
| KEGG | hsa05218 | Melanoma | 0.094772 | MITF, EGFR | 18.63014 | 0.959569 |
